# Roles of Macrophages in Viral Infections

**DOI:** 10.3390/v16101643

**Published:** 2024-10-21

**Authors:** Xiao-Long Wang, Xu Wang, Wen-Zhe Ho

**Affiliations:** Department of Pathology and Laboratory Medicine, Temple University Lewis Katz School of Medicine, Philadelphia, PA 19140, USA

Macrophages are an important component of the first-line defense against pathogens, including viruses. However, macrophages can be targeted by viral infections and can carry and spread viruses. The complicated roles of macrophages in antiviral immunity and the pathogenesis of viral infections warrant further studies. That is, to understand how macrophages interact with viral infections and play a dual role in the innate immune response by producing antiviral factors and inflammatory cytokines. This Special Issue thus presents seven original research articles [1,2,3,4,5,6,7] and two reviews [8,9], which provide further experimental evidence supporting the vital roles of macrophages in diverse viral infections, including in human immunodeficiency virus (HIV) -1/HIV-2 [3,5,7,8,9], hepatitis C virus (HCV) [6], influenza [4], Epstein–Barr virus (EBV) [2], severe acute respiratory syndrome coronavirus 2 (SARS-CoV-2) [6], and coxsackievirus B3 (CVB3) [1]. Using human peripheral blood monocyte-derived macrophages, mouse bone marrow-derived macrophages, and human-induced pluripotent stem cell (hiPSC)-derived macrophages (iMACs), the investigators studied the interactions between viral infections, bacteria product, and macrophage innate responses. Next, we briefly summarize the major findings of these seven research papers.

Wang et al. [1] showed that CVB3 infection inhibited NLRP3 inflammasome activation and IL-1β production in macrophages by suppressing the NF-κB signaling pathway and ROS production. They also observed that compared with uninfected animals, CVB3-infected mice had significantly lower expression of IL-1β and NLRP3 in the small intestine after LPS stimulation. Additionally, CVB3 infection increased the susceptibility of mice to Escherichia coli infection which was associated with decreased IL-1β production.

Moyano et al. [2] compared peripheral cytokine levels with macrophage polarization markers and viral protein expression in the tonsils of EBV-positive pediatric patients. Only IL-10 showed a negative correlation between compartments, and this was exclusively observed in patients undergoing EBV reactivation. Higher levels of peripheral IL-1β, IL-23, and IL-12p40 were observed in children (median age of 5 years). Lower levels of local and peripheral TNF-α were demonstrated in patients with broader expression of latent and lytic viral proteins as the EBV infection progressed. In healthy EBV carriers, IL-23 positively correlated with CD163, and IP-10 positively correlated with CD68. Their results indicated that EBV might modulate antigen expression in the presence of TNF-alpha and influence peripheral cytokine expression.

Gao et al. [3] reported that HIV-1 infection of human monocyte-derived macrophages (MDM) induced beta chemokine CCL2 expression. In contrast, HIV-2 infection decreased CCL2 expression in MDM. Their mechanistic studies showed that HIV-2 inhibited the expression of the STAT2, a key transcription factor for inducing CCL2. The blockade of STAT1 in HIV-infected MDM by a STAT1 inhibitor significantly reduced the production of CCL2. Furthermore, they showed that STAT1 reduction in HIV-2-infected MDM was regulated by the CUL2/RBX1 ubiquitin E3 ligase complex-dependent proteasome pathway. The knockdown of CUL2 or RBX1 restored the expression of STAT1 and CCL2 in HIV-2-infected MDM.

Yang et al. [4] reported that the extract of Scutellaria Baicalensis (SBE), a traditional Chinese herb medicine, could attenuate the influenza virus-elicited activation of the pattern recognition receptors (PRRs), TLR3/7/8, RIG-I/MDA5, NLRP3, and cGAS in macrophages. SBE inhibited the essential transcriptional genes in the PRR pathways and the nuclear translocation of NF-κB p65 in macrophages. These findings suggest that by suppressing macrophage activation by the virus, SBE has therapeutic potential for the clinical treatment of influenza virus infection.

Zhou et al. [5] examined the effect of flagellin, a bacteria product involved in HIV infection-mediated microbial translocation, on HIV-1 infection of primary human macrophages. They observed that the pretreatment of macrophages with flagellins from the different bacteria significantly inhibited HIV-1 infection. Mechanistic studies showed that flagellins downregulated the expression of the major HIV-1 entry receptors (CD4 and CCR5) and upregulated the CC chemokines (MIP-α, MIP-1β, and RANTES), and the ligands of CCR5. These effects of the flagellins could be counteracted by a toll-like receptor 5 (TLR5) antagonist. Because the flagellin has been commonly used as a vaccine adjuvant in TLR5 activation-mediated immune regulation, this study is clinically relevant. However, future investigations are necessary to determine the in vivo impact of flagellin–TLR5 interaction on macrophage-mediated innate immunity against HIV-1 infection and the effectiveness of flagellin adjuvant-based vaccines studies.

Zhang et al. [6] investigated the application of iMACs to study the interactions between macrophages and several human pathogens, including HCV, SARS-CoV-2, and Streptococcus pneumoniae. They concluded that iMAC is a valuable in vitro model to study the macrophage response to human viral and bacterial infections.

Kim et al. [7] used two EcoHIV clones encoding EGFP to infect primary murine brain cell cultures modeling NeuroHIV infection. They showed that EcoHIV could replicate efficiently in bone marrow-derived macrophages. In mixed brain cell cultures, EcoHIV targeted microglia but did not cause neuronal apoptosis. However, the productive infection could activate microglia and impair synaptophysin expression, dendritic density, and axonal structure in the brain, which contributed to cognitive dysfunctions. These findings indicate that this EcoHIV murine brain culture model is a valuable tool for the study of HIV neuropathogenesis.

Finally, this Special Issue also includes two review papers [8,9]. Woottum et al. provide a comprehensive review of the studies related to the critical functions (innate immunity/inflammation) of tissue macrophages in the pathophysiology of HIV-1 infection at various stages [8]. In the other review paper by Marra et al., the authors summarize current anti-HIV-1 therapies and highlight the role of macrophages as an HIV cellular reservoir, along with the most recent clinical studies on HIV disease [9].
